# Pathway-specific population attributable fractions

**DOI:** 10.1093/ije/dyac079

**Published:** 2022-05-10

**Authors:** Maurice M O’Connell, John P Ferguson

**Affiliations:** Biostatistics Unit, HRB Clinical Research Facility Galway, School of Medicine, NUI Galway, Galway, Ireland; Biostatistics Unit, HRB Clinical Research Facility Galway, School of Medicine, NUI Galway, Galway, Ireland

**Keywords:** Attributable fraction, risk factor, potential outcomes, causal inference

## Abstract

**Introduction:**

A population attributable fraction represents the relative change in disease prevalence that one might expect if a particular exposure was absent from the population. Often, one might be interested in what percentage of this effect acts through particular pathways. For instance, the effect of a sedentary lifestyle on stroke risk may be mediated by blood pressure, body mass index and several other intermediate risk factors.

**Methods:**

We define a new metric, the pathway-specific population attributable fraction (PS-PAF), for mediating pathways of interest. PS-PAFs can be informally defined as the relative change in disease prevalence from an intervention that shifts the distribution of the mediator to its expected distribution if the risk factor were eliminated, and sometimes more simply as the relative change in disease prevalence if the mediating pathway were disabled. A potential outcomes framework is used for formal definitions and associated estimands are derived via relevant identifiability conditions. Computationally efficient estimators for PS-PAFs are derived based on these identifiability conditions.

**Results:**

Calculations are demonstrated using INTERSTROKE—an international case–control study designed to quantify disease burden attributable to a number of known causal risk factors. The applied results suggest that mediating pathways from physical activity through blood pressure, blood lipids and body size explain comparable proportions of stroke disease burden, but a large proportion of the disease burden due to physical inactivity may be explained by alternative pathways.

**Conclusion:**

PS-PAFs measure disease burden attributable to differing mediating pathways and can generate insights into the dominant mechanisms by which a risk factor affects disease at a population level.

Key Messages

- A pathway-specific population attributable fraction (PS-PAF) measures the population disease burden attributable to a single risk factor→mediator→disease pathway.
- Comparing differing PS-PAFs can help in determining the dominant causal pathways by which a risk factor causes disease.
- A causal inference framework is used to define, identify and estimate PS-PAFs.
- Estimation of PS-PAFs for mediating relationships between physical activity and stroke suggests that pathways involving blood pressure, blood lipids and body size only partially explain stroke disease burden due to physical inactivity.

## Introduction

Population attributable fractions (PAFs) represent the relative change in disease prevalence that one might expect if a particular exposure was absent from the population. This metric was originally introduced in 1953 by Levin^1^ to estimate the percentage of lung cancer that would not have occurred under a counterfactual scenario in which nobody smoked in the population. Since then, these families of metrics have become a standard way of measuring total disease burden attributable to a risk factor^2^ and also to rank differing risk factors for prioritization as intervention targets.^3^

Partitioning this overall disease burden into contributions from the known pathways through which the risk factor affects disease is also useful both in understanding pathogenic mechanisms and also when comparing interventions that may reduce disease. For instance, we might estimate that in a hypothetical world, where dietary red meat was completely substituted for by plant-based protein, the prevalence of heart disease might be reduced by 10%. How much of this reduction in disease burden is attributable to the pathway by which diet affects blood pressure? Here, we introduce the pathway-specific population attributable fraction (PS-PAF) to help answer this question. In the preceding example, the PS-PAF can be informally understood as the relative change in disease prevalence in a hypothetical world in which the distribution of blood pressure was altered to match the distribution expected under the aforementioned dietary substitution. Under certain assumptions (described later), the same quantity can be described more mechanistically as the proportion of disease that would be avoided from completely disabling the corresponding mediating pathway at an individual level (here the pathway is diet→blood pressure→heart disease). PS-PAFs can be calculated for multiple mediating pathways for the same exposure. For example, the effect of the previous dietary substitution might be partially mediated by the effect on cholesterol as well as blood pressure; separate PS-PAFs could be compared for both pathways. In this case, the aim is not to provide an additive decomposition of the overall attributable fraction into PAFs for mediating pathways. Just as differing attributable fractions for a set of risk factors typically sum to more than the joint attributable fraction,^4^^,^^5^ differing PS-PAFs corresponding to various mediating pathways typically sum to more than the overall PAF for the risk factor. Rather than decomposing the total PAF, the aim instead is to fairly compare disease burden attributable to differing pathways and as a result gain insights into the dominant mechanisms by which the risk factor affects disease at a population level. These insights may in turn be useful in comparing possible interventions to prevent disease.

Presently, the only metric we know of in the literature that measures the extent of population disease burden through a risk factor→mediator→outcome pathway is indirect PAF, recently introduced by Sjölander.^6^ This metric operates by subtracting a direct attributable fraction (which corresponds to the disease burden contributed by all pathways not operating through the mediator) from the total PAF for the risk factor. However, as we explain later, the direct component will differ over differing mediators and so differing indirect PAFs over different mediators will be non-comparable. In this regard, the PS-PAF that we introduce here is more suited to measuring and comparing disease burden over differing known and unknown pathways. Our work is also related to the work of Vansteelandt and Daniel^7^ who examine pathway-specific effects of a treatment in a multi-mediator setting. A major difference between our work and theirs is that our results pertain to attributable fractions rather than individual-level treatment effects. Attributable fractions (such as the regular attributable fraction and the PS-PAF defined here) depend innately on the prevalence of a risk factor as well as relative risk and correspond to the proportion of disease burden attributable to the risk factor (as opposed to comparisons of the situations in which everyone and no one had the risk factor). As such, the PS-PAF could be regarded as the proportion of disease burden in the population related to a particular risk factor→mediator→outcome pathway, or alternatively how prominent that particular pathway is in the pathogenesis of the disease.

To maximize both the applicability and the interpretability of the described methods, we will define PS-PAFs under causal identifiability conditions of various stringency. Separable conditions are the most stringent and presume that an intervention is possible (at least in theory) to exactly replicate the effect of eliminating the risk factor on a particular mediator, with the intervention having no residual effect on disease that does not operate through the mediator. In this setting, PS-PAFs are associated with the effect of applying this hypothesized intervention at a population level. More applicable but perhaps less interpretable are the mechanistic and interventional forms that unlike the separable PS-PAFs require consideration of potential outcomes but can be applied when a separable intervention cannot be imagined. Fortunately, all three forms of the PS-PAF are identified in the same way so one can use the same estimation procedure and interpret depending on the assumptions they find reasonable. Having defined and identified PS-PAFs, we will compare and contrast them with the related concept of indirect PAFs introduced by Sjölander.^6^ We will illustrate results using data from INTERSTROKE—a case–control study designed to quantify disease burden attributable to a number of known causal risk factors for stroke. A discussion section concludes the manuscript.

## Identification and estimation of PS-PAFs

### Potential outcome notation used for mediation analyses

We borrow notation from VanderWeele^8^ in defining potential outcomes. As is usual in the causal inference literature, observable random variables will be denoted using unscripted notation and random variables for potential outcomes will be denoted using subscripts. In all cases, we use uppercase letters to denote random quantities and lowercase to denote quantities that are fixed or intervened on.

**Figure 1:**
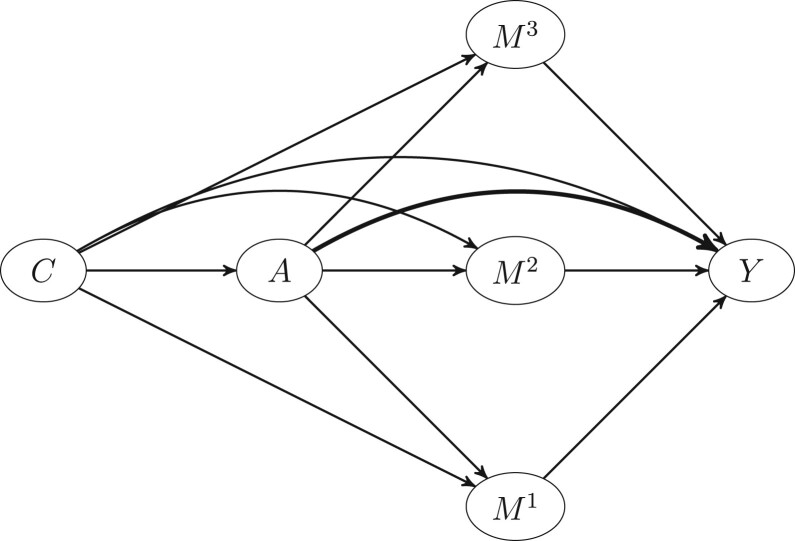
Directed acyclic graph (DAG) showing a causal structure linking exposure (*A*), mediators (*M*^1^, *M*^2^, *M*^3^), confounders (*C*) and outcome (*Y*)

In particular, let *C* denote a random vector of known baseline covariates not affected by the exposure, the random variable A∈{0,1} a binary exposure of interest and the random variables $M^{1},\ldots M^{K}$ mediators on separate causal pathways from *A* to *Y*; each *M^k^* could be binary, multi-category or continuous. Finally the random variable Y∈{0,1} represents a binary disease outcome. Figure 1 demonstrates a multi-mediator scenario with three mediators: *M*^1^, *M*^2^ and *M*^3^.

The potential outcome setting exposure to *a* and *M^k^* to *m^k^* is denoted by $Y_{a,m^{k}}$, where the subscript *k* indicates which mediator is intervened on. On occasion, we will consider potential outcomes setting the exposure to *a* and the *K* mediators to $m^{1},\ldots ,m^{K}$, denoted by $Y_{a,m^{1},\ldots ,m^{K}}$. Since an intervention setting *A *=* *0 may well affect the distribution of *M^k^*, the potential outcome $Y_{a,m^{1},\ldots ,m^{K}}$ for particular individuals may be unrealizable even under plausible interventions on the risk factor. However, under standard counterfactual models, such as Pearl’s NPSEM-IE (Non-Parametric Structural Equations Model with Independent Errors), the quantity $Y_{a,m^{1},\ldots ,m^{K}}$ is still well defined for these individuals and can be utilized when defining causal effects. One can also define potential outcomes for each *M^k^*, k≤K for an intervention setting the exposure to *a* as $M_{a}^{k}$. In addition, we will use the following abbreviated notation: $Y_{a}=Y_{a,M_{a}^{1},\ldots ,M_{a}^{K}}$ for an intervention that sets the exposure *A* to *a*, $Y_{A,m^{k}}$ (corresponding to an intervention setting the *k*th mediator to *m^k^*, with the exposure taking its natural value) and $Y_{0,M^{k}}$ for an intervention ‘eliminating’ the exposure but where the *k*th mediator takes its natural value. Note that implicit in this notation is the possibility that an intervention on the *k*th mediator might affect causally downstream mediators when several mediators lie on the same causal pathway. If on the other hand the mediators lie on separate causal pathways that are d-separated given *A* and *C* (briefly, this means that in the causal graph relating *C*, *A*, *M*^1^,..,*M^K^*, any path of adjoining arrows starting from *M^j^* and ending at *M^k^* that do not pass through *Y* must intersect either *A* or *C*), then $Y_{A,m^{k}}$ = $Y_{A,m^{1},\ldots ,m^{K}}$, where $m^{j}=M^{j}$ for j≠k, *M^j^* being the observed value of the *j*th mediator under no intervention, and $Y_{0,M^{k}}$=$Y_{0,m^{1},\ldots ,m^{K}}$, where $m^{k}=M^{k}$ and $m^{j}=M_{0}^{j}$ for $j\neq k,\;M_{0}^{j}$ being the value that the *j*th mediator would take under an intervention that sets the exposure to 0. A summary of all of this notation is given in Summary Box 1.

**Table:** **Summary Box 1** Potential outcome notation used in the manuscript

C	Known baseline confounders
A∈{0,1}	Binary exposure or risk factor
Y∈{0,1}	Binary indicator for disease
$M^{1}$ ,…,$M^{K}$	K≥1 known mediators of A→Y
$Y_{a}$	Potential outcome for Y setting A=a
$M_{a}^{k}$	Potential outcome for $M^{k}$, k≤K setting A=a
$Y_{a,m^{k}}$	Potential outcome for Y, setting A=a and $M^{k}=m^{k}$
$Y_{a,m^{1},...,m^{K}}$	Potential outcome for Y setting A=a, $M^{1}=m^{1},\ldots ,\;M^{K}=m^{K}$

As is usual with causal inference using the potential outcomes framework, we make Stable Unit Treated Value Assumptions (SUTVA):^9^ the relationship between potential and observed outcomes satisfy ‘consistency’, which implies for instance that $Y=Y_{a}=Y_{a,M_{a}^{1},\ldots ,M_{a}^{K}}$ when *A *=* a*. In addition, mediation analysis requires some technical conditional independence assumptions that we will list as needed in the following sections.

### The PAF

Despite being an intrinsically causal idea, attributable fractions were originally defined via conditional probabilities in a non-causal framework, which has contributed to confusion regarding what they really purport to measure. Here, we will instead use a definition based on potential outcomes that is now becoming more prominent:^10^^,^^11^

(1) $$PAF_{\mathrm{total}}=\frac{P(Y=1)-P(Y_{0}=1)}{P(Y=1)}.$$

Thinking of *Y*_0_ as the potential outcome for a random individual in a population in which no one was exposed to the risk factor, Equation (1) can be directly interpreted as the relative change in disease prevalence if that risk factor was absent from the population.

### The PS-PAF

Whereas *PAF_total_* measures the total disease burden attributable to a risk factor, PS-PAF measures disease burden attributable to a risk factor→mediator→outcome pathway. We will give three differing causal definitions for PS-PAFs with interventional, mechanistic and separable interpretations. We start by defining the interventional pathway-specific PAF, which is most generally applicable, and as shown later reduces to the mechanistic and separable forms when additional assumptions are met.

For a binary risk factor A∈{0,1}, the interventional PS-PAF for the mediating pathway *A*→*M^k^*→*Y* is defined as:

(2) $$PAF_{A->M^{k}->Y}^{I}=\frac{P(Y=1)-P(Y_{A,G_{0|C}^{k}}=1)}{P(Y=1)}$$

where the random variable $G_{0|C}^{k}$ is a random draw from the conditional distribution of $M_{0}^{k}$ given an individual’s covariates, *C*. Marginally, $G_{0}^{k}$ (simulated via first randomly sampling an individual from the population and using their covariates, *C*, to generate $G_{0|C}^{k}$) is a draw from the population distribution of $M_{0}^{k}$, i.e. the distribution that *M^k^* would have in a hypothetical world in which the risk factor was eliminated. Noting this, the PS-PAF can then be interpreted in terms of a population intervention on *M^k^*, where its distribution is shifted to the distribution that would be observed in a population in which the risk factor *A* was eliminated, with other quantities in the population (covariates *C*, exposure *A* and other mediators $M^{j},j\neq k$, which are not causally downstream of *M^k^*) remaining unchanged. This idea of randomly assigned interventions has previously been introduced to estimate versions of natural direct and indirect effects (sometimes termed interventional direct and indirect effects) in the presence of exposure-induced confounding.^7^^,^^12^

One might also want to compare the disease burden attributable to pathways that are unknown (or involve unobserved mediators). To do this, we will adapt the concept of the direct PAF proposed by Sjölander^6^ as follows:

(3) $$PAF_{A->Y}^{M^{1},\ldots ,M^{K}}=\frac{P(Y=1)-P(Y_{0,M^{1},\ldots ,M^{K}}=1)}{P(Y=1)}$$

We will refer to Equation (3) as the PS-PAF for the direct pathway *A*→*Y*. Equation (3) measures the disease burden attributable to direct pathogenic pathways from *A* to *Y*, i.e. any pathway from *A* to *Y* (that may or may not be known) excluding the set of mediating pathways under consideration. See Table 1 for a summary of the interpretations of Equations (1), (2) and (3).

It is instructive to compare the interpretations of direct and PS-PAFs in a more concrete setting. In the introduction we informally illustrated PS-PAFs in the context of the effect of a dietary substitution (which was red meat being completely substituted for by plant-based proteins). In this case the PS-PAF through blood pressure could be informally defined as the relative change in disease prevalence in a world in which the distribution of blood pressure was somehow altered to match the distribution expected under such a population-level substitution but without changing the dietary habits of the population. In contrast the direct PS-PAF would imagine the relative change in disease prevalence in a second world in which the dietary substitution was rigorously implemented at a population level but the effect of this intervention on blood pressure as well as on other known mediators (e.g. body mass index) was disabled so the individual-level values of all these mediators were unaltered.

The definition by Sjölander^6^ is very similar except that it refers to the direct pathway with reference to a single mediating pathway, *k*, and will change dependent on that mediating pathway as follows:

(4) $$PAF_{A->Y}^{M^{k}}=\frac{P(Y=1)-P(Y_{0,M^{k}}=1)}{P(Y=1)}$$

In the previous example, this direct definition (with reference to blood pressure) compares prevalence in a world in which the dietary substitution was implemented and as before the effect of this intervention on blood pressure is disabled but with the values of other known mediators of the diet/CVD relationship (where CVD is cardiovascular disease) now affected by the intervention. In the case that there is only a single mediating pathway, Equations (3) and (4) will coincide.

### Identifiability conditions

As is usual in mediation analysis, causal identifiability conditions need to hold to estimate Equation (2) in an unbiased way from data (the data comprising observed values of covariates, exposure, mediator and response sampled from some population). In particular, Conditions (i) and (ii) below are necessary to identify PS-PAFs for each mediator *M^k^* of interest:

-
$M_{0}^{k}⊥⊥A|C$ (i.e. conditional on covariate strata, the potential outcome for the mediator, under elimination of the risk factor, and the natural value of the risk factor should be independent random variables. This condition can be informally understood as associations between the risk factor and mediator having causal interpretations within strata of covariates).
-
$Y_{a,m^{k}}⊥⊥M^{k}|A=a,C$ (i.e. conditional on covariate and risk factor strata, potential outcomes for the response, under various assignments for risk factor and the *k^th^* mediator, and the natural value of *M^k^* should be independent. Informally, this can be understood as associations between the *k*th mediator and outcome having causal interpretations within joint strata of risk factor and covariates). An additional condition is necessary to identify $PAF_{A->Y}$
-
$Y_{a,M^{1},\ldots ,M^{K}}⊥⊥A|M^{1},\ldots ,M^{K},C$ [i.e. conditional on joint strata of covariates and mediators *M*^1^,…,*M^K^*, the potential outcome $Y_{a,M^{1},\ldots ,M^{K}}$ and risk factor *A* are independent random variables; informally this can be understood as associations between the risk factor and outcome having causal interpretations within joint strata of mediators ($M^{1},\ldots ,M^{K}$) and covariates].

Note that under identifiability Conditions (i), (ii) and (iii), we show in the Supplementary material (available as Supplementary data at *IJE* online) that:

(5) $$\begin{array}{c} PAF_{A->M^{k}->Y}^{I}=\frac{P(Y=1)-E_{A,C}(E_{M^{k}|A=0,C}(P(Y=1|A,C,M^{k})))}{P(Y=1)} \end{array}$$

and

(6) $$\begin{array}{c} PAF_{A->Y}=\frac{P(Y=1)-E_{C,M^{1},\ldots ,M^{K}}(P(Y=1|A=0,C,M^{1},\ldots ,M^{K}))}{P(Y=1)} \end{array}$$

where for generic functions *g*_1_, *g*_2_ and *g*_3_, $E_{A,C}(g_{1}(A,C))=\int_{a,c}g_{1}(a,c)dF_{A,C}(a,c)$ and $E_{C,M^{1},\ldots ,M^{K}}g_{2}(C,M^{1},..,M^{K})=\int_{c,m^{1},\ldots ,m^{K}}g_{2}(c,m^{1}\ldots ,m^{K})dF_{C,M^{1},\ldots ,M^{K}}(c,m^{1},\ldots ,m^{K})$ represent expectations of the random variables $g_{1}(A,C)$ and $g_{2}(C,M^{1},\ldots ,M^{K})$, integrated over the marginal distributions $F_{A,C}$ and $F_{C,M^{1},\ldots ,M^{K}}$ of the subscripted variables and $E_{M^{k}|A=0,c}(g_{3}(M^{k}))=\int_{m^{k}}g_{3}(m^{k})dF_{M^{k}|A=0,C=c}(m^{k})$ is an expectation of $g_{3}(M^{k})$ integrated according to the conditional distribution of *M^k^* given *A *=* *0 and *C *=* c*, $F_{M^{k}|A=0,C=c}$. [Note also the shorthand that is used in the notation for conditional distributions of *Y* and *M^k^*, e.g. a probability like $P(Y=1|A,C,M^{k})$ stands for the probability that *Y *=* *1 given the risk factor takes the value *A*, confounders take the value *C* and the *k*th mediator takes the value *M^k^*, where *A*, *C* and *M^k^* are thought of as random. When evaluating this probability at fixed values *C *=* c*, $M^{k}=m^{k}$ and *A *=* a*, we instead write: $P(Y=1|A=a,C=a,M^{k}=m^{k})$.]

### Mechanistic PS-PAFs and disabling pathways

Under an extra identifiability condition, the pathway-specific PAF can be also expressed in a mechanistic form where the mediator assignment to an individual (within the hypothetical population where the distribution of the mediator is altered) is the mediator that would result for that individual under no exposure to the risk factor:

(7) $$\begin{array}{c} PAF_{A->M^{k}->Y}^{M}=\frac{P(Y=1)-P(Y_{A,M_{0}^{k}}=1)}{P(Y=1)} \end{array}$$

This final condition is:

iv.  $Y_{a,m^{k}}⊥⊥M_{0}^{k}|A=a,C$.

**Figure 2:**
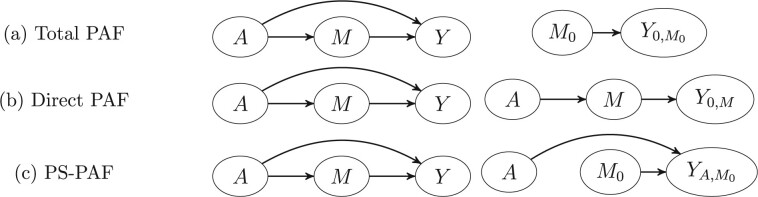
Illustration of total, direct and pathway-specific population attributable fraction (PS-PAF) as a comparison between disease risk in the current population (graph on left-hand side) and counterfactual disease risk in a population in which particular pathways through which the risk factor affects disease are disabled (graph on right-hand side). A situation with a single mediated pathway: *A*→*M*→*Y* is illustrated. Confounders, *C*, are committed for display purposes. (a) Total PAF compares observed disease risk, *P*(*Y* = 1), vs disease risk, *P*(*Y*_0,__*M*__0_ = 1) in a hypothetical population in which both direct and mediated pathways are disabled. (b) Direct PAF compares observed disease risk vs disease risk *P*(*Y*_0,__*M*_ = 1) in a hypothetical population in which the direct pathway is disabled. (c) Pathway-specific PAF compares observed disease risk *Y* vs disease risk *P*(*Y_A_*_,__*M*__0_ = 1) in a hypothetical population in which the mediated pathway *A*→*M*→*Y* is disabled.

This condition is less intuitive than Conditions (i), (ii) and (iii) described in the preceding section and involves consideration of cross-world counterfactuals (i.e. if *a *=* *1, then $Y_{a,m^{k}}$ and $M_{0}^{k}$ would never be observed on the same individual). However, as shown in the Supplementary material (available as Supplementary data at *IJE* online), this condition does hold in a non-parametric structural equations model provided there is no post-treatment confounding of the mediator–outcome relationship. The mechanistic pathway-specific PAF can be thought of as the relative change in disease burden in a hypothetical population in which the mediated pathway *A*→*M^k^*→*Y* is disabled. For example, in a simple setting in which there is only a single known mediator, *M*, there are two potential pathways by which the risk factor affects disease, represented by the pathways *A*→*M*→*Y* and *A*→*Y* in Figure 2a. The total PAF (which refers to a population in which a binary risk factor was eliminated) corresponds to the disabling of both pathways, i.e. the comparison is of disease risk in the populations with causal graphs shown in the left-hand and right-hand panes of Figure 2a. In contrast, direct PAF involves a comparison of disease risk in the current population with the hypothetical population in which the direct pathway is disabled (Figure 2b) whereas the pathway-specific PAF represents a comparison of the current population with a hypothetical population in which the pathway *A*→*M*→*Y* is disabled (Figure 2c). Note that in each case, current disease risk is compared with disease risk in some hypothetical population in which a particular pathway (or pathways) has been disabled.

### Alternative framework using separable paths

**Figure 3:**
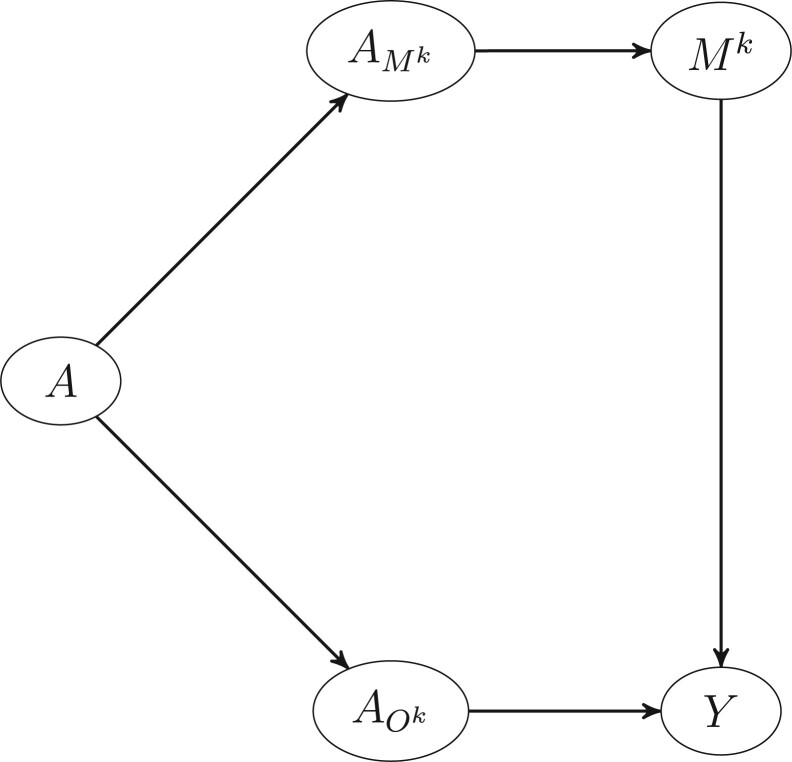
Extended graph representing a situation in which an intervention on the risk factor *A* can be separated into two components, each component representing differing mechanisms by which *A* might affect *Y*. The component $A_{M}^{k}$ only affects *Y* through its relationship with the mediator *M^k^*, whereas $A_{O}^{k}$ has a causal effect on *Y* possibly through other mediators but not through *M^k^*. If this framework is appropriate, we need to imagine at least hypothetical interventions setting $A_{M}^{k}$ or $A_{O}^{k}$ to particular values independently of each other. For simplicity, confounding variables, *C*, which would be represented in the graph by a root node *C* pointing to *A*, *M^k^* and *Y*, have been omitted.

An alternative interpretation of pathway-specific PAFs may be obtained using the separable paths framework detailed in Section 5 of Robins and Richardson.^13^ Under this framework for mediation, we imagine that the effect of a treatment *A* can be split into two (or more) components, each component representing differing mechanisms by which A might affect Y. For example, in the directed acyclic graph (DAG) represented by Figure 3, component $A_{M^{k}}$ only affects *Y* through its direct relationship with the mediator *M^k^*, whereas $A_{O^{k}}$ affects *Y* either directly or through mediators other than *M^k^*. In the real world, $A_{M^{k}}$ and $A_{O^{k}}$ are linked deterministically based on the value of *A*, e.g. if *A *=* a*, then $A_{M^{k}}=a$ and $A_{O^{k}}=a$ with probability 1 for each a∈{0,1}. However, if this framework is appropriate, interventions need to be conceived of that can set component $A_{M^{k}}$ to a particular value independently of the value of $A_{O^{k}}$. Whether this is appropriate needs to be judged on a case-by-case basis. For example, perhaps we are interested in the PS-PAF for physical activity on stroke through blood pressure. If it is plausible that the effect of increased physical activity on blood pressure could be replicated by taking an antihypertensive pill with the pill having no direct effect on stroke that is not mediated through blood pressure, assignment of the antihypertensive pill may represent an intervention that sets $A_{M^{k}}$= 0 independently of the value of $A_{O^{k}}$ and the PS-PAF could be defined as in Equation (8) below. In contrast, there is no obvious intervention replicating the effect of physical activity on waist–hip ratio with no casual effect on stroke that is not mediated through the waist–hip ratio. As a result, defining the PS-PAF for paths from physical inactivity through the waist–hip ratio via Equation (8) may not be appropriate. More formally, the separable PS-PAF is defined as:

(8) $$PAF_{A->M^{k}->Y}^{S}=\frac{P(Y=1)-P(Y=1|do(A_{M^{k}}=0))}{P(Y=1)}$$

where here we use the do notation, popularized by Pearl^14^ to represent the ‘interventional’ distribution where $A_{M^{k}}$ is set to 0 in the population. In analogy with how attributable fractions are usually defined, the above represents the relative change in the probability of disease in a population in which the ‘component’ risk factor $A_{M^{k}}$ was set to 0 in the population but with the distribution of $A_{O^{k}}$ remaining unchanged (effectively disabling the pathway *A*→*M^k^*→*Y*). More specifically, suppose that risk factor A∈{0,1} is divisible into separable components $A_{M^{k}}$ and $A_{O^{k}}$, with causal DAG as represented in Figure 3, with $A=A_{M^{k}}=A_{O^{k}}$ with probability 1. Assuming that 0<P(A=0|C=c) for all possible values of the covariate vector *c*, we prove in the Supplementary appendix (available as Supplementary data at *IJE* online) that:

$$P(Y=1|do(A_{M^{k}}=0)=E_{A,C}(E_{M^{k}|A=0,C}(P(Y=1|A,C,M^{k}))),$$

indicating that the same identification formula results under interventional, mechanistic and separable interpretations. These three interpretations are compared in Table 2.

### PS-PAFs vs indirect PAFs

**Figure 4:**
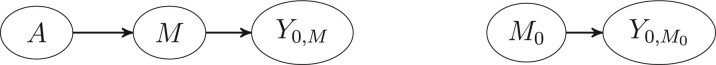
An indirect population attributable fraction compares disease risk *P*(*Y*_0,_*_M_* = 1) in a hypothetical population with the direct pathway disabled with the disease risk *P*(*Y*_0,__*M*__0_ = 1) in a hypothetical population in which both direct and mediated pathways have been disabled

In the context of a single mediating pathway, Sjölander^6^ introduced the concept of indirect PAFs:

(9) $$\begin{array}{c} PAF_{\mathrm{indirect},M^{k}}=PAF_{\mathrm{total}}-PAF_{A->Y}^{M^{k}} \end{array}$$

defined so that it sums with $PAF_{A->Y}^{M^{k}}$, in Equation (4), to the total PAF. Rather than comparing disease risk in the current and hypothetical populations, Equation (8) implicitly compares disease risk in two hypothetical populations: one in which the direct pathway *A*→*Y* has been disabled, with a second in which the direct and mediated pathways are both disabled (see Figure 4). An indirect PAF will usually be smaller than the corresponding pathway-specific PAF in a one mediator situation, as it is likely that some disease cases in the current population that are exposed to the risk factor might be equally well prevented by either eliminating the effect of the direct pathway (i.e. perhaps $Y_{1,M_{1}}=1$, but $Y_{0,M_{1}}=0$) or eliminating the mediated pathway (i.e. perhaps $Y_{1,M_{1}}=1$, but $Y_{1,M_{0}}=0$). The prevention of such disease cases would contribute to the pathway-specific PAF but not to the indirect PAF. See Table 3 for a comparison of the differences between pathway-specific and indirect population attributable fractions.

### Estimating PS-PAFs

We will denote values for the risk factor $A_{i}\in \{0,1\}$, covariate vector *C_i_* and mediators $M_{i}^{1},\ldots ,M_{i}^{K}$ for each individual i≤N in the data set. Estimation in cohort and cross-sectional studies is more straightforward than in case–control studies since the components of Equations (5) and (6) can in theory be consistently estimated using empirical conditional distributions for the observed variables in our data. In contrast, case–control studies cannot be regarded as random or representative samples from the overall population and the corresponding estimated empirical distributions will be biased for their population counterparts. However, if the prevalence of disease, *π*, is known and the cases and controls are randomly selected from their source populations, one can weight the contributions of each individual i≤N so that the reweighted sample is representative of the source population and consistent estimation of distributional quantities from the population is feasible. If the case:control matching ratio is 1:*r* for some r≥1, this implies weights *w_i_* = 1 for cases and $w_{i}=(1/\pi -1)/r$ for controls. For cohort and cross-sectional studies, we define *w_i_* = 1 for all i≤N.

We first describe the estimator when *M^k^* is continuous. We suppose that the researcher specifies and estimates models for $P(Y=1|A=a,C=c,M^{k}=m)$ and $E(M^{k}|A=c,C=c)$ (perhaps using the reweighted data set in case–control scenarios). For each individual *i*, we define ${\hat{M}}_{i}^{k}=M_{i}^{k}-A_{i}(\hat{E(M_{i}^{k}|A_{i}=1,C_{i})}-\hat{E(M_{i}^{k}|A_{i}=0,C_{i}))}$. Then our estimator for PS-PAF is:

$${\hat{\mathrm{PAF}}}_{A->M^{k}->Y}=\frac{\sum w_{i}Y_{i}-\sum_{i}w_{i}\hat{P(Y=1|A_{i},C_{i},{\hat{M}}_{i}^{k}})}{\sum w_{i}Y_{i}}$$

If *M^k^* is discrete, having a finite number of levels $M^{k}=\{m_{1},\ldots ,m_{n_{k}}\}$, we need to estimate the probabilities $P(M^{k}=m|A_{i}=0,C_{i})$ for each $m\in M^{k}$, in which case the estimator for PS-PAF is as follows:

$${\hat{\mathrm{PAF}}}_{A->M^{k}->Y}=\frac{\left[\sum w_{i}Y_{i}-\sum_{i}w_{i}\sum_{m\in M^{k}}\hat{P(M^{k}=m|A_{i}=0,C_{i})}\hat{P(Y=1|A_{i},C_{i},M^{k}=m)}\right]}{\sum w_{i}Y_{i}}$$

In the case in which there are multiple mediators, an inconvenience in applying the above estimators for differing mediators $M^{1},\ldots ,M^{K}$ is separate outcome models: $\hat{P(Y=1|A=a,C=c,M^{k}=m^{k})}$ need to be fit for separate mediators, *M^k^*, k≤K. Here, we assume the mediators lie on separate causal pathways that are d-separated given *A* and *C* (or alternatively *M*^1^,..,*M^K^* are mutually conditionally independent given *A* and *C*) in which case it follows that:

$$\begin{array}{c} E_{A,C}(E_{M^{k}|A=0,C}(P(Y=1|A,C,M^{k})))\\ =E_{A,C,M^{\neq k}}(E_{M^{k}|A=0,C}(P(Y=1|A,C,M^{1},\ldots ,M^{K}))) \end{array}$$

letting $M^{\neq k}$ be the set of mediators excluding *M^k^*, which implies that a single outcome model $\hat{P(Y=1|A=a,C=c,M^{1}=m^{1},\ldots ,M^{K}=m^{K})}$ can be used, suggesting the alternative estimators:

$${\hat{\mathrm{PAF}}}_{A->M^{k}->Y}=\frac{\sum w_{i}Y_{i}-\sum_{i}w_{i}\hat{P(Y=1|A_{i},C_{i},{\hat{M}}_{i}^{k},M_{i}^{\neq k})}}{\sum w_{i}Y_{i}}$$

and


$${\hat{\mathrm{PAF}}}_{A->M^{k}->Y}=\frac{\left[\sum w_{i}Y_{i}-\sum_{i}w_{i}\sum_{m\in M^{k}}\hat{P(M^{k}=m|A_{i}=0,C_{i})}\hat{P(Y=1|A_{i},C_{i},M^{k}=m,M_{i}^{\neq k})}\right]}{\sum w_{i}Y_{i}},$$

where $M_{i}^{\neq k}$ are the set of mediator values for individual *i*, excluding mediator *k*. In the alternative case in which mediators lie on the same causal pathway (with some mediators acting as confounders for other mediator–outcome relationships), Condition (ii) will be violated for those *M^k^* subject to confounding by other mediators and alternative estimation approaches that are described in the Supplementary material (available as Supplementary data at *IJE* online) in the section titled “Identification of interventional pathway-specific population attributable fractions (PS-PAF) with more general structural dependence between multiple mediators involving post treatment confounding” need to be used. Justifications of these estimators including the modelling assumptions necessary for consistent estimation are given in the Supplementary material (available as Supplementary data at *IJE* online). Since these estimators combine models for *M^k^* and *Y*, analytic formulae for standard errors are difficult to derive. In the data example given below, we instead calculate approximate confidence intervals for pathway-specific PAFs using the bootstrap (see Table 4). In contrast, estimating $PAF_{A->Y}$ is more straightforward and boils down to estimating $E_{C,M^{1},\ldots ,M^{K}}(P(Y=1|A=0,C,M^{1},\ldots M^{K})))$. This can be achieved by estimating $P(Y=1|A=0,C_{i},M_{i}^{1},\ldots ,M_{i}^{K})$ for each individual and then averaging this quantity over individuals in the data, taking care to incorporate weighting under a case–control design. As an alternative, a double robust estimator for $E(P(Y=1|A=0,C,M^{1},\ldots ,M^{K}))$ can be derived using the same approaches as Sjölander describes.^6^

**Table 1:** Interpretations for direct and pathway-specific population attributable fractions (PAFs)

Metric	Formula	Interpretation
$PAF_{A->Y}^{M^{1},...,M^{K}}$	$\frac{P\left(Y=1\right)-P\left(Y_{0,M_{1},...,M_{K}}=1\right)}{P\left(Y=1\right)}$	Relative decrease in disease prevalence in hypothetical population in which risk factor has been eliminated but without affecting the distribution of any of the known mediators
$PAF_{A->M^{k}->Y}^{I}$	$\frac{P\left(Y=1\right)-P\left(Y_{A,G_{0|C}^{k}}=1\right)}{P\left(Y=1\right)}$	Relative decrease in disease prevalence in hypothetical population in which $M^{k}$ is distributed as if the risk factor *A* was eliminated but the distribution of *A* itself is unaffected
$PAF_{\mathrm{total}}$	$\frac{P\left(Y=1\right)-P\left(Y_{0}=1\right)}{P\left(Y=1\right)}$	Relative decrease in disease prevalence in hypothetical population in which risk factor *A* has been removed

$PAF_{A->Y}^{M^{1},...,M^{K}}$ represents the direct PAF, $PAF_{A->M^{k}->Y}^{I}$ represents the interventional version of the pathway-specific attributable fraction for the pathway *A*→*M^k^*→*Y* $\mathrm{and}\;PAF_{\mathrm{total}}$ represents the total PAF. In each case the disease prevalence in the current population is compared with prevalence in a hypothetical population in which a particular intervention is enforced.

**Table 2:** Comparison of interventional: $PAF_{A->M^{k}->Y}^{I},\;\mathrm{mechanistic}:\;PAF_{A->M^{k}->Y}^{M}\;\mathrm{and}\;\mathrm{separable}:\;PAF_{A->M^{k}->Y}^{S}$ forms for the pathway-specific population attributable fraction (PS-PAF)

	$PAF_{A->M^{k}->Y}^{I}$	$PAF_{A->M^{k}->Y}^{M}$	$PAF_{A->M^{k}->Y}^{S}$
Assumptions	$M_{0}^{k}⊥A|C$ $Y_{{a,m}^{k}}\;⊥{M\;}^{K}|\;A=a,\;C$	$M_{0}^{k}⊥A|C$ $Y_{{a,m}^{k}}\;⊥M^{k}|\;A=a,\;C$ $Y_{{a,m}^{k}}\;⊥M_{0}^{k}|\;A=a,\;C$	A can be represented as the composition of two independently manipulable variables, $A_{M^{k}}$ and $A_{O^{k}}$. The intervention setting $A_{M^{k}}=0$ is equivalent to disabling the mediating pathway *A*→*M^k^*→*Y* (see Figure 3)
Interpretation	Relative decrease in disease prevalence in hypothetical population in which $M^{k}$ is distributed as if the risk factor A was eliminated (given covariates *C*) but the distribution of A itself is unaffected	Relative decrease in disease prevalence in hypothetical population in which the mediating pathway *A*→*M^k^*→*Y* is disabled at an individual level	The PAF corresponding to an intervention that sets the component $A_{M^{k}}$ of A to 0 at a population level. Treating $A_{M^{k}}$ as a risk factor in its own right, this is a standard attributable fraction
+Ve/–Ve	+Ve: most generally applicable (identifiable under the most general conditions). Note that mechanistic and separable PS-PAFs are also interventional PS-PAFs –Ve: Perhaps least interpretable	+Ve: Interpretation in terms of disabling mediating pathway at an individual level –Ve: Identification requires cross-world assumptions	+Ve: Interpretation as a regular attributable fraction –Ve: Limited applicability due to separability requirement for A

**Table 3:** Comparison between the pathway-specific population attributable fraction (PS-PAF) and the indirect population attributable fraction (PAF)

	$PAF_{A->M^{k}->Y}^{I}$	$PAF_{\mathrm{indirect},M^{k}}$
**Interpretation**	Relative decrease in disease prevalence in hypothetical population in which $M^{k}$ is distributed as if the risk factor A was eliminated but the distribution of A itself is unaffected	Remainder after subtracting [$PAF_{A->Y}^{M^{k}}$ [Equation (4)] from $PAF_{\mathrm{total}}$. Can be interpreted as a sequential PAF for disabling the pathway *A*→*M^k^*→*Y* subsequent to disabling all pathways from *A*→*Y* not mediated by $M^{k}$
**Additivity**	Summing PS-PAF over differing mediators with direct PAF does not equal total PAF	$PAF_{A->Y}^{M^{k}}+PAF_{\mathrm{indirect},M^{k}}=PAF_{\mathrm{total}}$ for each *k.* However, differing indirect PAFs (referring to separate pathways) will not sum to total PAF
**Relative size**	Provided mediating pathways are deleterious, will be larger than indirect PAF	Usually will be smaller than corresponding PS-PAF
**Comparability with direct PAF**	$PAF_{A->Y}^{M^{1},...,M^{K}}$ [Equation (3)] is actually a type of PS-PAF corresponding to all unobserved and unknown pathways (from *A*→*Y*) and is directly comparable with $PAF_{A->M^{k}->Y}$	Not directly comparable with Equation (3) due to its interpretation as a sequential PAF for the mediating pathway having disabled all pathways not mediated by $M^{k}$

$PAF_{A->M^{k}->Y}^{I}\;\mathrm{represents}\;\mathrm{the}\;\left(\mathrm{interventional}\right)\;\mathrm{PS}-\mathrm{PAF}\;\mathrm{for}\;\mathrm{the}\;\mathrm{pathway}\;A\to M^{k}\to Y\;\;\mathrm{and}\;PAF_{\mathrm{indirect},M^{k}}$ the indirect population attributable fraction for $M^{k}$. Given that the interventional PS-PAF is most generally applicable (i.e. the PS-PAF can always be interpreted in this way), we focus on the interventional PS-PAF in this comparison.

**Table 4:** Results from estimating the pathway-specific, direct and indirect population attributable fractions (PAFs) on INTERSTROKE risk factors

Pathway	Total PAF	$PAF_{A->Y}^{M^{1},...,M^{K}}$	$PAF_{A->Y}^{M^{k}}$	$PAF_{\mathrm{Indirect},M^{k}}$	$PAF_{A->M^{k}->Y}$
PHYS→HBP→STROKE	0.40 (0.35, 0.46)	0.34 (0.27, 0.41)	0.38 (0.32, 0.44)	0.025 (0.001, 0.049)	0.042 (0.015, 0.069)
PHYS→WHR→STROKE	0.40 (0.35, 0.46)	0.34 (0.27, 0.41)	0.39 (0.33, 0.45)	0.017 (–0.001, 0.035)	0.028 (0.017, 0.038)
PHYS→APOB→STROKE	0.40 (0.35, 0.46)	0.34 (0.27, 0.41)	0.39 (0.33, 0.45)	0.016 (–0.003, 0.035)	0.025 (0.003, 0.045)

$PAF_{A->M^{k}->Y}\;\mathrm{represents}\;\mathrm{the}\;\mathrm{pathway}-\mathrm{specific}\;\mathrm{population}\;\mathrm{attributable}\;\mathrm{fraction}\;\left(\mathrm{PS}-\mathrm{PAF}\right),\;PAF_{A->Y}^{M^{1},...,M^{K}}t\mathrm{he}\;\mathrm{direct}\;\mathrm{PAF},\;PAF_{\mathrm{Indirect},M^{k}}\;\mathrm{the}\;\mathrm{indirect}\;\mathrm{PAF}.\;\mathrm{PHYS}\;\mathrm{codes}\;\mathrm{for}\;\mathrm{physical}\;\mathrm{inactivity}\;\left(\mathrm{yes}\;\mathrm{or}\;\mathrm{no}\right),\;\mathrm{HBP}\;\mathrm{for}\;\mathrm{diagnosed}\;\mathrm{hypertension}\;\left(\mathrm{yes}\;\mathrm{or}\;\mathrm{no}\right),\;\mathrm{WHR}\;\mathrm{for}\;\mathrm{waist}–\mathrm{hip}\;\mathrm{ratio}\;\left(\mathrm{measured}\;\mathrm{continuously}\right)a\mathrm{nd}\;\mathrm{APOB}\;\mathrm{for}\;\mathrm{the}\;\mathrm{ratio}:\mathrm{apo}-\mathrm{lipoprotein}\;A/\mathrm{apo}-\mathrm{lipoprotein}\;B,\;\mathrm{again}\;\mathrm{measured}\;\mathrm{continuously}.\;\mathrm{Sj}ö\mathrm{lander}’s\;\mathrm{form}\;\mathrm{of}\;\mathrm{the}\;\mathrm{direct}\;\mathrm{PAF}:\;PAF_{A->Y}^{M^{k}}\;\mathrm{is}\;\mathrm{also}\;\mathrm{given}\;\mathrm{for}\;\mathrm{comparison}\;\mathrm{purposes}.$ Estimated 95% CIs (estimate±1.96*SE) are shown in brackets. Standard errors (SEs) were estimated using 200 bootstrap iterations. PAF estimates are rounded to two significant digits.

## Data example

**Figure 5:**
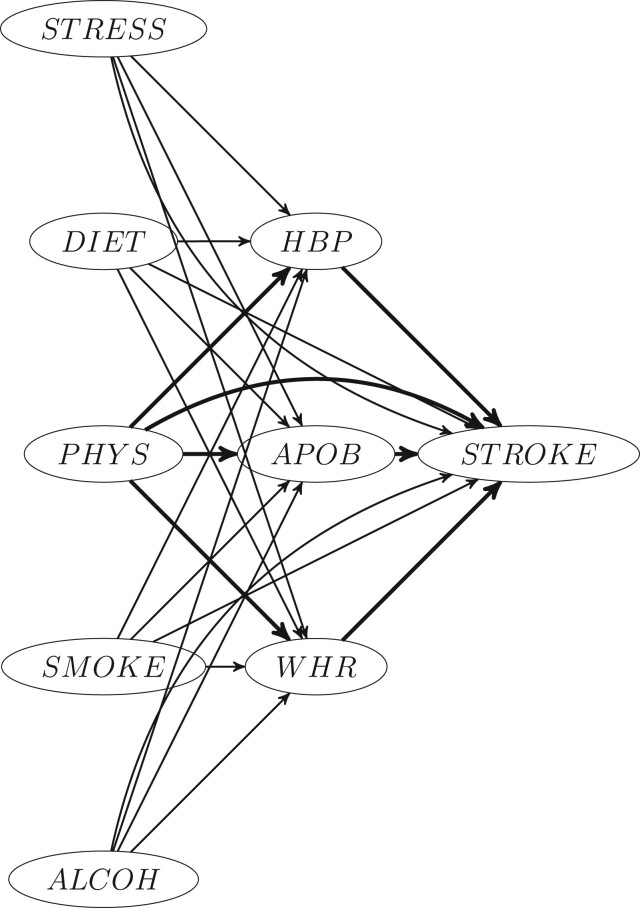
Direct acyclic graph showing the assumed causal structure for risk factors in INTERSTROKE used in analyses. Risk factor codings are as follows. STRESS: psychological stress, DIET: healthy eating index, PHYS: physical inactivity (a binary variable), SMOKE: tobacco consumption, ALCOH: alcohol consumption, HBP: a binary indicator for high blood pressure, APOB: ratio between Apo-lipoproteins A and B, WHR: waist–hip ratio, STROKE: binary indicator for whether the patient suffered a stroke. Arrows for direct and mediating pathways associated with physical inactivity are shown in bold font. Age, sex, education level and geographic region are confounders for the risk factor/disease relationship for all listed risk factors on the figure (these are omitted for display purposes).

INTERSTROKE^15^ is a large international case–control study designed to quantify the contribution of established risk factors to stroke prevalence at a global level. Here we investigate the possible mediating effects of physical inactivity (PHYS: a two-level variable with levels ‘mainly inactive’ coded as 1 and mainly active coded as 0) on incidence of first stroke through waist–hip ratio (WHR) [waist measurement (cm)]/[hip measurement in (cm)], ratio between measured apolipoprotein-B and apolipoprotein-A (APOB) (these are proteins in the blood responsible for lipid metabolism) and prior clinical diagnosis of high blood pressure (HBP). We treat WHR and APOB as continuous variables and HBP as binary. Covariates and assumed mediators are as assumed in the causal structure shown in Figure 5.

To estimate Sjölander’s direct and indirect attributable fractions, and the PS-PAFs described above, we fit a main-effects logistic regression predicting stroke status as a function of age, sex, region, education, healthy-eating score, self-reported stress levels, smoking status, alcohol use, PHYS, WHR, APOB and HPB, with the terms for WHR and APOB entering as 5-degree of freedom natural cubic splines to ensure sufficient flexibility to model the speculated non-linear relationships between these mediators and stroke. The covariates were chosen with the hope that relationships between mediators (APOB, WHR and HBP) and the outcome might be close to unconfounded conditional on physical activity and the other covariates (as required in condition (ii)). Stroke controls were upweighted by a factor of 284 to reflect a yearly stroke incidence of first stroke of 0.0035 or 3.5 strokes per 1000 individuals per year, estimated via data from the global burden of disease.^16^ Models for each mediator are conditioned on age, sex, region, education, healthy-eating score, stress levels, smoking status, alcohol use and PHYS. These covariates (excluding PHYS) are assumed to to act as a sufficient adjustment set to deconfound the relationship between PHYS and each mediator with the hope that this set of covariates satisfies condition (i).

In this example, the mediating pathways and direct pathway due to physical inactivity (here A = 1 represents individuals that are physically inactive, A = 0 represents individuals who are physically active) are likely to all increase the probability of stroke, and one would expect PS-PAFs to be somewhat larger than the corresponding indirect PAFs. This pattern is seen in Table 4 with the estimated PS-PAFs all being larger than the corresponding indirect PAFs. As an example, the PS-PAF for HBP, which is interpreted as the relative decrease in disease prevalence if one disabled the physical activity→HBP→stroke pathway is estimated to be 4.2% (one might informally interpret this as the percentage of stroke burden that is attributable to this pathway). The indirect PAF, interpretable as the relative decrease in disease prevalence associated with disabling the same mediating pathway, but now subsequent to disabling all pathways from physical inactivity to stroke that don’t involve HBP is estimated to be 2.5%. Similarly, the estimated PS-PAFs through APOB (2.5%) and waist-hip ratio (2.8%) are larger than the corresponding indirect PAFs (1.6%) and (1.7%). The direct $PAF_{A->Y}^{M^{1},...,M^{K}}$ (defined in Equation (3)) is estimated as 34% and represents the relative decrease in disease prevalence if all pathways not mediated by HBP, APOB or WHR were disabled. In Table 4, we also report estimates for Sjölander’s version of direct PAF: $PAF_{A->Y}^{M^{K}}$ given by Equation (4), which changes depending on the mediating pathway (essentially this estimates disease burden through all other pathways except through M^k^). In summary, this analysis suggests that population disease burden for stroke attributable to physical inactivity partially depends on the mediating pathways through blood pressure, WHR and APOB but depends mostly on other (unknown) mechanisms, exemplified by the large direct PAF. As with any causal analysis, these tentative conclusions depend jointly on correct modelling of conditional probability distributions and on the validity of the causal identifiability assumptions listed earlier.

## Discussion

In this paper we have introduced PS-PAFs (in interventional, mechanistic and separable forms) as a metric to measure the disease burden attributable to particular exposure mediator pathways. Whereas at first sight this triplicate of definitions seems to add complexity, it should be noted that all three metrics are identified the same way in observational data (but under differing assumptions) and in some way represent a sequence of generalizations of separable PS-PAFs. For instance, in contrast to the interventional and mechanistic versions, the separable PS-PAF is identifiable without a counterfactual model and counterfactual notation is unnecessary in its definition. However, if we associate a typical counterfactual model to the causal DAG in Figure 3 (such as Pearl’s Non-Parametric Structural Equations Model with Independent Errors (NPSEM-IE)^17^ or Robins’s Finest Fully Randomised Causally Interpretable Structural Tree Graph (FFRCISTG) for one step forward counterfactuals^18^), $P(Y=1|do(A_{M^{k}}=0))$ will correspond to $P(Y_{A,M_{0}^{k}}=1)$ (see the Supplementary material, available as Supplementary data at *IJE* online, for more justification). In other words, if the separable PS-PAF is identifiable then so is the mechanistic PS-PAF and the two will correspond. Similarly, if assumptions (i), (ii) and (iv) are true (the assumptions necessary to identify the mechanistic PS-PAF), then certainly assumptions (i) and (ii) are both true and the interventional PS-PAF can be identified (and since the identification formula is the same, they must again correspond). Indeed, as we show in the Supplementary material (available as Supplementary data at *IJE* online), the interventional PS-PAF is identifiable under more general structural relationships between the mediators (compared with the mechanistic and separable PS-PAFs) where certain mediators of the risk factor–outcome relationship act as confounders for the relationship between other mediators and the outcome. In contrast, the mechanistic and separable pathway-specific PAFs are not identifiable in situations involving post-treatment confounding. Although the least identifiable, the separable PS-PAF has perhaps the cleanest interpretation both in terms of a PAF for disabling the mediating pathway and also as a regular PAF for $A_{M^{k}}$ (i.e. treating the separable component $A_{M^{k}}$ of *A* as a risk factor). Likewise the interpretation of the mechanistic PS-PAF (in terms of disabling the mediating pathway) is more palatable than the interventional PS-PAF (in terms of an intervention in which each individual is assigned a random value of the mediator from its distribution under elimination of the risk factor, conditional on their covariates).

PS-PAFs are related to the ideas of direct and indirect PAF recently proposed by Sjölander.^6^ Whereas PS-PAFs have an interpretation that compares disease prevalence in the actual and hypothetical populations, indirect PAFs do not and are defined as the leftover disease burden after subtracting the direct PAF from the total PAF. As such, the interpretation of indirect PAFs is intrinsically linked to direct PAFs and is perhaps difficult to motivate in a clinical setting. For instance, in a situation in which the direct PAF and PS-PAF are both 100%, disease could be eliminated by disabling either the direct pathway or alternatively disabling the mediating pathway. Despite the possibility of eradicating disease by disabling the mediating pathway, the indirect PAF is defined as 0%. In a one mediator situation, one way to understand the distinctions between these concepts is in terms of modified sequential attributable fractions,^4^ i.e. attributable fractions that are constructed from disabling pathways in a particular order (as demonstrated in Figures 2 and 4) with the order in which pathways are disabled differing for PS-PAFs and indirect PAFs. In more detail, a (mechanistic) PS-PAF as given by Equation (7) can be interpreted as the relative change in disease burden from disabling a particular mediating pathway. The corresponding indirect PAF, Equation (9), is also associated with disabling that same mediating pathway but this time subsequent to disabling the direct pathway (see Figure 4). Since the effect of disabling both the direct and mediating pathways is equivalent to the effect of eliminating the risk factor, this effectively forces the additivity property that the total PAF is the sum of the direct and indirect PAFs. Note that in general, sequential attributable fractions^19^ are constructed to sum to some well-defined overall PAF but usually the sequence corresponds to the hypothetical elimination of each of a group of risk factors in some order rather than disabling mediating pathways related to the effect of a particular risk factor in some order. Whereas this additivity property at first seems appealing, it perhaps is unnatural in the context of attributable fractions, where it is well recognized that the PAF for differing risk factors may sum to more than the joint PAFs and sometimes to >1.^20^ The sufficient/component cause framework^21^ gives a simple but enlightening explanation for this phenomenon. For particular individuals, a certain collection of risk factors (perhaps diet, stress and tobacco usage) might collectively lead to disease at a particular point in time but the disease may not have occurred at that time if any of the risk factors were not present. The same logic implies that pathway-specific PAFs will tend to be larger than indirect PAFs, as illustrated in this manuscript, if the direct and indirect pathways act as independent disease-causing mechanisms both of which need to be operational in particular individuals for disease to occur. If additivity to the total PAF is required while at the same time preserving the comparability of measured disease burden for direct and mediating pathways, it is possible to average sequential analogues of PS-PAFs to produce a kind of average PS-PAF that, when summed over differing mediating and direct pathways, equals the total PAF. This, however, is beyond the scope of the paper and as a quantity may be difficult to interpret.

Although, throughout this manuscript, we imagine that a mediator is observed at a point in time, often longitudinal exposure to the mediator (e.g. sustained HBP over several years due to a sedentary lifestyle) is an important contributor to disease risk. Whereas it is relatively easy to extend definitions of PS-PAFs to reflect counterfactual mediator trajectories, identifiability conditions are more complicated and longitudinal data on mediators and associated time-varying confounders are then necessary for estimation. Nevertheless, even if longitudinal data on mediator progression are lacking, as they are here, estimating PS-PAFs for differing pathways as calculated here can act as a proxy for a longitudinal calculation and still help in determining the dominant pathways by which a risk factor affects an outcome. For instance, the analysis here suggests that the pathways from physical inactivity through the waist–hip ratio, blood pressure and cholesterol may have reasonably similar contributions to disease burden, and suggests that much of the disease burden due to inactivity is explained by alternative pathways.

Although attributable fractions can measure the total disease burden associated with a risk factor, they are less useful to measure the real-world impact of a public health intervention on that risk factor since even successful health interventions usually only partially eliminate a risk factor and in addition cannot alter prior history to a risk factor when cumulative exposure might also impact disease. As an example, rather than considering a hypothetical population in which smoking is eliminated, a realistic population-level intervention (such as increasing the tax on cigarettes) may result in a 5% decrease in the number of cigarettes consumed rather than total elimination of smoking. Impact fractions are generalized versions of attributable fractions that measure the reduction in disease prevalence associated with such a population intervention.^22^ The ideas described here can easily be adapted to define and estimate pathway-specific impact fractions for such real-world interventions that may characterize the dominant mechanisms by which the intervention affects disease burden. For example, the pathway-specific impact fraction for the pathway *A*→*M^k^*→*Y* could be defined by letting $G_{|C}^{k}$ represent a random variable having the population distribution of the mediator *M^k^* under the proposed intervention (simulated again conditional on an individuals’ covariate vector *C*) and replacing $G_{0|C}^{k}$ with $G_{|C}^{k}$ in Equation (2).

## Ethics approval

No human patients were directly involved in this study. The research involves secondary analysis of anonymized data and ethics approval was not required.

## Supplementary Material

dyac079_Supplementary_Data

## Data Availability

The INTERSTROKE data set used in the real data example is not publicly available. Simulated case–control data for INTERSTROKE risk factors based on a fitted Bayesian network model applied to the real INTERSTROKE data, as well as examples of calculating PS-PAFs using this simulated data, are available in the R-package graphPAF: https://github.com/johnfergusonNUIG/graphPAF.
