# Supplementary material for: Pathway-specific population attributable fractions
Source: Int J Epidemiol. 2022 May 10;51(6):1957–69. doi: 10.1093/ije/dyac079 (PMC9749703; doi:10.1093/ije/dyac079)
Supplement: dyac079_Supplementary_Data [file dyac079_supplementary_data.zip › dyac079_Supplementary_Data/ije-2021-10-1464-File012.pdf]

Pathway-specific population attributable fractions

Supplementary information

Maurice M O'Connell[1] and John P Ferguson[*]

[1]Biostatistics Unit, HRB Clinical Research Facility Galway, NUI Galway, Ireland
[*]Corresponding author: John Ferguson, Biostatistics Unit, HRB Clinical Research Facility Galway, NUI Galway, Ireland.
Email: john.ferguson@nuigalway.ie

Identification of interventional pathway-specific population attributable fraction (PS-PAF) under conditions 1 and 2.

The following is a proof of the identifiability formula for pathway-specific attributable fractions, using assumptions 1. and 2. detailed in the main manuscript. In the following and subsequent derivations, we assume that covariates, C and mediators $M^1, ..., M^K$ have discrete distributions. The general cases of mixed discrete and continuous random variables with a well defined joint distribution follows similarly by replacing summations with integrals over general probability measures.

$P(Y_{A,G^j_{0|C}} = 1)$

$$= \sum_{c,a,m^j} P(Y_{a,G^j_{0|C}} = 1|A = a, C = c, G^j_{0|C} = m^j)P(C = c)P(A = a|C = c)P(G^j_{0|C} = m^j|C = c, A = a)$$

$$= \sum_{c,a,m^j} P(Y_{a,m^j} = 1|A = a, C = c, G^j_{0|C} = m^j)P(C = c)P(A = a|C = c)P(M^j_0 = m^j|C = c)$$

$$= \sum_{c,a,m^j} P(Y_{a,m^j} = 1|A = a, C = c)P(C = c)P(A = a|C = c)P(M^j_0 = m^j|C = c)$$

$$= \sum_{c,a,m^j} P(Y_{a,m^j} = 1|A = a, C = c)P(C = c)P(A = a|C = c)P(M^j_0 = m^j|A = 0, C = c)$$

$$= \sum_{c,a,m^j} P(Y_{a,m^j} = 1|A = a, C = c, M^j = m^j)P(C = c)P(A = a|C = c)P(M^j_0 = m^j|A = 0, C = c)$$

$$= \sum_{c,a,m^j} P(Y = 1|A = a, C = c, M^j = m^j)P(C = c)P(A = a|C = c)P(M^j = m^j|A = 0, C = c)$$

$$= E_{A,C}(E_{M^j|A=0,C}(P(Y = 1|A, C, M^j)))$$

The first equality follows from the double expectation theorem. The second equality follows from the definition of $G^j_{0|C}$ (which is generated from the distribution of M^j_0 conditional on C, independently of A) and the 3rd equality since conditional on A and C, $G^j_{0|C}$ is independent of Y_{a,m^j}. The fourth equality follows as $M^j_0 \perp\!\!\!\perp A|C$. The fifth equality follows since $Y_{a,m} \perp\!\!\!\perp M|A = a, C$. The sixth equality follows by consistency.

Identification of mechanistic pathway-specific population attributable fraction (PS-PAF) under conditions 1, 2 and 4.

$P(Y_{A,M_0^j}=1)$

$$= \sum_{c,a,m^j} P(Y_{a,M_0^j}=1|A=a,C=c,M_0^j=m^j)P(C=c)P(A=a|C=c)P(M_0^j=m^j|A=a,C=c)$$

$$= \sum_{c,a,m^j} P(Y_{a,m^j}=1|A=a,C=c,M_0^j=m^j)P(C=c)P(A=a|C=c)P(M_0^j=m^j|A=a,C=c)$$

$$= \sum_{c,a,m^j} P(Y_{a,m^j}=1|A=a,C=c)P(C=c)P(A=a|C=c)P(M_0^j=m^j|A=a,C=c)$$

$$= \sum_{c,a,m^j} P(Y_{a,m^j}=1|A=a,C=c)P(C=c)P(A=a|C=c)P(M_0^j=m^j|A=0,C=c)$$

$$= \sum_{c,a,m^j} P(Y_{a,m^j}=1|A=a,C=c,M^j=m^j)P(C=c)P(A=a|C=c)P(M_0^j=m^j|A=0,C=c)$$

$$= \sum_{c,a,m^j} P(Y=1|A=a,C=c,M^j=m^j)P(C=c)P(A=a|C=c)P(M^j=m^j|A=0,C=c)$$

$$= E_{A,C}(E_{M^j|A=0,C}(P(Y=1|A,C,M^j)))$$

Here the proof is almost the same the proof of the pathway-specific PAF. The main difference is the cross world assumption independence assumption: $Y_{a,m^j} \perp\!\!\!\perp M_0^j|A=a,C$ is needed to reduce $P(Y_{a,m^j}=1|A=a,C=c,M_0^j=m^j)$ to $P(Y_{a,m^j}=1|A=a,C=c)$ in the third equality. In contrast, in the previous argument, the equality $P(Y_{a,m^j}=1|A=a,C=c,G_{0|C}^j=m^j)$=$P(Y_{a,m^j}=1|A=a,C=c)$, follows since $G_{0|C}^j$ is randomly generated conditional on C and as a result is independent of Y_{a,m^j} conditional on A and C.

Identification of $PAF_{A->Y}$ under condition 3.

$P(Y_{0,M^1,..,M^K}=1)$

$$= \sum_{c,m^1,...,m^K} P(Y_{0,M^1,...,M^K}=1|C=c,M^1=m^1,...,M^K=m^K)P(C=c)P(M^1=m^1,...,M^K=m^K|C=c)$$

$$= \sum_{c,m^1,...,m^K} P(Y_{0,M^1,...,M^K}=1|C=c,M^1=m^1,...,M^K=m^K,A=0)P(C=c)\times$$
$P(M^1=m^1,..,M^K=m^K|C=c)$

$$= \sum_{c,m^1,...,m^K} P(Y=1|C=c,M^1=m^1,...,M^K=m^K,A=0)P(C=c)P(M^1=m^1,...,M^K=m^K|C=c)$$

$$= E_{C,M^1,...,M^K}(P(Y=1|A=0,C,M^1,...,M^K))$$

The first equality follows from iterated expectation theorem. Here the 3rd identifiability condition: $Y_{0,M^1,...,M^K} \perp\!\!\!\perp A|M^1,...,M^K,C$ is used to show $P(Y_{0,M^1,...,M^K}=1|C=c,M^1=m^1,...,M^K=m^K)$=$P(Y_{0,M^1,...,M^K}=1|C=c,M^1=m^1,...,M^K=m^K,A=0)$ in the second equality. The final equality follows from consistency.

Proof of equivalence of individual mediator and joint mediator approaches to estimation when mediators are conditionally independent given exposure and confounders

In the Estimation section of the main manuscript we stated that:

$$E_{A,C}(E_{M^1|A=0,C}(P(Y=1|A,C,M^1)))$$

$$= E_{A,C,M^2,...,M^K}(E_{M^1|A=0,C}(P(Y=1|A,C,M^1,...,M^K)))$$

, where we set $j=1$ in M^j for simplicity of notation, assuming the mediators are jointly independent conditioned on the values of exposure, A and confounders C.

We will prove this statement by first proving two simpler assertions (1):

$$P(Y=1|A=a,C=c,M^1=m^1)$$

$$= E_{M^2,...,M^K|A=a,C=c}P(Y=1|A=a,C=c,M^1=m^1,M^2,...,M^K)$$

for any values of exposure a and confounders c, and (2):

$$E_{M^1|A=0,C=c}(g(M^1)) = E_{M^1|A=0,C=c,M^2=m^2,...,M^K=m^k}(g(M^1)).$$

for any $m^2,...,m^K$.

To prove assertion (1):

$$P(Y=1|A=a,C=c,M^1=m^1)$$

$$= \sum_{m^2,...,m^K} P(Y=1,M^2=m^2,...,M^K=m^K|A=a,C=c,M^1=m^1)$$

$$= \sum_{m^2,...,m^K} P(Y=1|M^1=m^1,M^2=m^2,...,M^K=m^K,A=a,C=c)P(M^2=m^2,...,M^K=m^K|A=a,C=c,M^1=m^1)$$

$$= \sum_{m^2,...,m^K} P(Y=1|M^1=m^1,M^2=m^2,...,M^K=m^K,A=a,C=c)P(M^2=m^2,...,M^K=m^K|A=a,C=c)$$

$$= E_{M^2,...,M^K|A=a,C=c}(P(Y=1|A=a,C,M^1=m^1,M^2,...,M^K))$$

Here the first equality holds due to the law of total probability, the second equality by properties of conditional and joint probabilities and the third

equality via conditional independence of the mediators given $A = a$ and $C = c$.

Assertion (2) follows since the probabilities measures $dF_{M^1|A=a,C=c}$ and $dF_{M^1|A=a,C=c,M^2=m^2,...,M^K=m^K}$ are equal due to conditional independence of the mediators.

Given these two assertions:

$$E_{A,C}(E_{M^1|A=0,C}(P(Y=1|A,C,M^1))$$

$$= E_{A,C}(E_{M^1|A=0,C}E_{M_2,...,M^K|A,C}P(Y=1|A,C,M^1,M^2,...,M^K)$$

$$= E_{A,C,M_2,....,M_K}E_{M^1|A=0,C}P(Y=1|A,C,M^1,M^2,...,M^K)$$

$$= E_{A,C,M_2,....,M_K}E_{M^1|A=0,C,M^2,...,M^K}P(Y=1|A,C,M^1...,M^K)$$

The first equality follows from assertion 1. For the 2rd equality, we use the identity:

$$E_{A,C}E_{M_2,...,M^K|A,C}g(M^2,...,M^K,A,C) = E_{A,C,M_2,...,M^K}g(M^2,...,M^K,A,C)$$

which follows from the iterated expectation theorem. The last equality follows from assertion 2.

Justification for estimation algorithm for pathway-specific population attributable fractions (PS-PAF)

First, we treat the case of a continuous mediator, M^k. For the estimation algorithm to be consistent we need to assume:

- The model for $P(Y=1|M^k=m^k, A=a, C=c)$ is correctly specified as a function of strata for mediator m^k, risk factor a and confounders c, and a estimator: $P(Y=1|M^k \widehat{= m^k}, A=a, C=c)$ in estimating these probabilities (that is uniformly statistically consistent over covariate, risk factor and mediator strata).

- A model for the mediator $E(M^k|A=a, C=c)$ and estimator $E(M^k|\widehat{A=a}, C=c)$ is used that has the property that $\hat{\theta(c)}= E(M^k|\widehat{A=1}, C=c) - E(M^k|\widehat{A=0}, C=c)$ is uniformly consistent for $\theta(c)= E(M^k|A=1, C=c) - E(M^k|A=0, C=c)$ across covariate strata.

- The function $P(Y=1|A=a, M^k=m^k, C=c)$ is uniformly continuous as a function of m^k across strata of A and C.

- Within each covariate strata, c, the distribution of the residual term $\epsilon_{k,c} = M^k - E(M^k|A=a, C=c)$ is invariant in a

Consider selecting an individual from the population at random, their values of covariates, risk factor, mediator and outcome being denoted C, A, M^k and Y. Let $\hat{M}^k = M^k$ if $A=0$ and $\hat{M}^k = M^k - (E(M^k|A=1, C) - E(M^k|A=0, C))$ when $A=1$. Suppose first $A=0$, then conditioning on A and C,

$$E_{\hat{M}^k|A,C}P(Y=1|A, C, \hat{M}^k)$$

$$= \int P(Y=1|A, C, m^k)dF_{\hat{M}^k|A=0,C}(m^k)$$

$$= \int P(Y=1|A, C, m^k)dF_{M^k|A=0,C}(m^k)$$

$$= E_{M^k|A=0,C}(P(Y=1|A, C, M^k)$$

since $\hat{M}^k = M^k$ when $A=0$, $F_{m^k|A=0,C}$ being the distribution function of M^k conditioned on A=0 and C. When $A=1$,

$$E_{\hat{M}^k|A,C}P(Y=1|A, C, \hat{M}^k)$$

$$= \int P(Y=1|A, C, m^k)dF_{\hat{M}^k|A=1,C}(m^k).$$

However, since conditional on $C = c$, the residual term $\epsilon_{k,c}$ is equi-distributed over different levels of a, it follows that

$$dF_{\hat{M}^k|A=1,C} = dF_{M^k|A=0,C}$$

as distribution functions, so again when $A = 1$,conditioning on C,

$$E_{\hat{M}^k|A,C}P(Y = 1|A, C, \hat{M}^k)$$

$$= \int P(Y = 1|A, C, m^k)dF_{M^k|A=0,C}(m^k)$$

$$= E_{M^k|A=0,C}(P(Y = 1|A, C, M^k)).$$

This argument then shows that selecting an individual at random, $P(Y = 1|A, C, \hat{M}^k)$ has mean

$$E_{A,C}E_{\hat{M}^k|A,C}P(Y = 1|A, C, M^k) = E_{A,C}E_{M^k|A=0,C}P(Y = 1|A, C, M^k)(*)$$

It follows that if individuals $i = 1, ..., N$ are a random sample of the population, $\sum P(Y = 1|A, C, \hat{M}^k)/N$ is an unbiased estimate of $E_{A,C}E_{\hat{M}^k|A,C}P(Y = 1|A, C, \hat{M}^k)$.

In reality though $\hat{M}^k = M^k - A(E(\widehat{M^k|A = 1}, C) - E(\widehat{M^k|A = 0}, C))$ and we estimate $P(Y = 1|A, C, m^k)$ with $P(Y = \widehat{1|A, C}, m^k)$. The uniform consistency and continuity assumptions allow us to plug in these estimated models into the estimator into (*) and still achieve a consistent estimator. Note that the approach has a robustness property that it will provide correct estimation of the PS-PAF even under incorrectly specified mediator models, provided the contrast $\theta(C) = E(M^k|A = 1, C) - E(M^k|A = 0, C)$ is correctly estimated over covariate strata C. [1]

In the discrete mediator case, the argument is more direct, by noting that:

$$E_{M^k|A=0,C}(P(Y = 1|A, C, M^k))$$

[1]Regarding this robustness property, note that estimating $\theta(C)$ is likely easier than estimating $E(M^k|A = a, C)$. For instance if the estimator $E(\widehat{M|A = a}, C)$ has some bias B that doesnt depend on a, then this bias cancels out of $\theta(\hat{c}) = E(\widehat{M|A = 1}, C)$-$E(\widehat{M|A = 0}, C)$ which is then unbiased for $\theta(C)$. Even with partial cancelation of the bias, $\theta(\hat{c})$ will be a less biased estimator than $E(\widehat{M|A = a}, C)$. In addition $\theta(C)$ is likely to vary less as a function of C than $E(M|A = a, C)$, and may be estimated with less variance. In particular, if there are no interactions between A and C in their effect on M, $\theta(C)$ is constant. In this case we can use the entire dataset to estimate $\theta(C)$, whereas only data around $C = c$ may be relevant in estimating $E(M|A = a, C = c)$.

$$= \sum_{m \in \mathcal{M}^k} P(M^k = m | A_i = 0, C_i) P(Y = 1 | A_i, C_i, M^k = m).$$

Provided uniformly consistent estimates of the models $P(M^k = m|A_i = 0, C_i)$ and $P(Y = 1|A_i, C_i, M^k = m)$ are available, we can substitute these into the preceding equation, and the estimator in the main manuscript will be consistent for the PS-PAF for M^j.

Non parametric structural equations and the validity of the cross world assumption, assuming no post treatment confounding

Under the assumption that the joint distribution of (C, A, M, Y) follows a non-parametric structural model, the cross world condition: $Y_{a,m} \perp\!\!\!\perp M_0 | A = a, C$ is satisfied. For simplicity of notation, we describe a one mediator situation in what follows; a similar argument can be used to demonstrate the same result when there are $K > 1$ mediators (this is provided the structural equations for each mediator depend only on A and C and not other mediators). Effectively the non-parametric structural equations model implies the joint distribution is generated sequentially from unknown deterministic functions F_C, F_A, F_M, F_Y as follows:

$$C = F_C(U_C)$$
$$A = F_A(C, U_A)$$
$$M = F_M(C, A, U_M)$$
$$Y = F_Y(C, A, M, U_Y)$$

where, U_C, U_A, U_M and U_Y are independent noise random variables that add stochasticity to the joint distribution. Potential outcomes can be easily derived using the above equations. For instance, conditioning on A=a and C,

$M_0 = F_M(C, 0, U_M)$ is a function of U_M, since $C = F_C(U_C)$ is conditioned on. Also $Y_{a,m} = F_Y(C, a, m, U_Y)$ is a function of U_Y, again since C is conditioned on. Since U_M and U_Y are independent, $Y_{a,m} \perp\!\!\!\perp M_0 | A = a, C$.

Note that the preceding argument breaks down if there is a confounder affected by exposure as below.

Mediator-outcome confounder affected by exposure

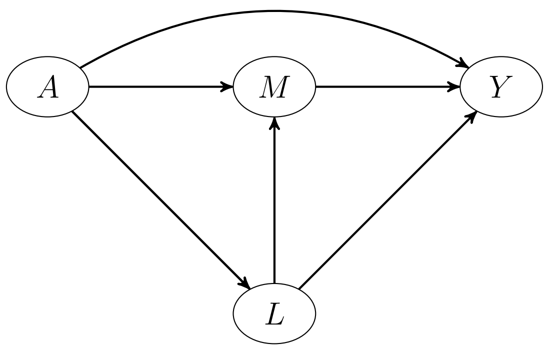

Figure 1: A DAG representing mediation of the effect of the exposure A on the outcome Y by the mediator M, with a variable L that is a mediator-outcome confounder affected by exposure. The presence of this "intermediate confounder" means the Mechanistic PS-PAF of A on Y through M is not non-parametrically identified, even in a non-parametric structural equation model with independent errors. In contrast, the interventional PS-PAF specified in equation 2 of the main manuscript is non-parametrically identified in such a model.

Identification of interventional pathway-specific population attributable fractions (PS-PAF) with more general structural dependence between multiple mediators involving post treatment confounding

In general, suppose we are interested in mediator M^j which is subject to post treatment confounding by the set of variables L (possibly involving other mediators) and data on L has been recorded. To be more exact, variables in L are affected by the risk factor A (represented in a causal graph with an arrow from A to L) but are also joint causes of M^j and Y (see Figure 1). Such a variable is termed as a post-treatment confounder or a recanting witness. [1].

In the identifiability conditions for interventional pathway-specific PAFs, we have assumed that $M^j \perp\!\!\!\perp Y_{a,m^j}|A = a, C$ for each j. This condition only allows certain structural dependencies between the mediators, and will be violated under post-treatment confounding. For instance, in the DAGs shown in Figures 2(b) and 2(c) this condition is violated for M^2 (b) and M^1 (c) due to post-treatment confounding (the variable L being represented by the other mediator in each case).

In fact, under post treatment confounding, the separable and mechanistic PS-PAFs are no longer identifiable without additional assumptions that don't hold in typical counterfactual models (see [2] for a related discussion). However, provided the identifiability condition (2*) given below is true:

$$(2^*) : Y_{a,m^j} \perp\!\!\!\perp M^j|A = a, C, L$$

and in addition condition (1): $M_0^j \perp\!\!\!\perp A|C$ from the main manuscript is also met, the interventional PS-PAF is still identified via the new formula:

$$PAF^I_{A->M^j->Y} = \frac{P(Y = 1) - E_{A,C,L}(E_{M^j|A=0,C}(P(Y = 1|A, C, M^j, L)))}{P(Y = 1)}.$$

(Note here that the variable L is suppressed in the counterfactual notation: Y_{a,m^j} might also be written as $Y_{a,L_a,m}$, in which case (2*) would instead be written as $Y_{a,l,m^j} \perp\!\!\!\perp M^j|A = a, C, L = l$, with minor changes to notation in the proof below). Note that the result follows in straightforward fashion provided we can show that $P(Y_{A,G^j_{0|C}} = 1) = E_{A,C,L}(E_{M^j|A=0,C}(P(Y = 1|A, C, M^j, L)))$. We prove this below.

$$P(Y_{A,G^j_{0|C}} = 1)$$

$$= \sum_{c,a,l,m^j} P(Y_{a,G^j_{0|C}} = 1|A = a, C = c, G^j_{0|C} = m^j, L = l)P(C = c)$$
$$P(A = a|C = c)P(L = l|A = a, C = c)P(G^j_{0|C} = m^j|C = c, A = a, L = l)$$

$$= \sum_{c,a,l,m^j} P(Y_{a,m^j} = 1|A = a, C = c, G^j_{0|C} = m^j, L = l)P(C = c)$$
$$P(A = a|C = c)P(L = l|A = a, C = c)P(M^j_0 = m^j|C = c)$$

$$= \sum_{c,a,l,m^j} P(Y_{a,m^j} = 1|A = a, C = c, L = l)P(C = c)$$
$$P(A = a|C = c)P(L = l|A = a, C = c)P(M^j_0 = m^j|C = c)$$

$$= \sum_{c,a,l,m^j} P(Y_{a,m^j} = 1|A = a, C = c, L = l)P(C = c)$$
$$P(A = a|C = c)P(L = l|A = a, C = c)P(M^j_0 = m^j|A = 0, C = c)$$

$$= \sum_{c,a,l,m^j} P(Y_{a,m^j} = 1|A = a, C = c, M^j = m^j, L = l)P(C = c)$$
$$P(A = a|C = c)P(L = l|A = a, C = c)P(M^j_0 = m^j|A = 0, C = c)$$

$$= \sum_{c,a,l,m^j} P(Y = 1|A = a, C = c, M^j = m^j, M = l)P(C = c)$$
$$P(A = a|C = c)P(L = l|A = a, C = c)P(M^j = m^j|A = 0, C = c)$$

$$= E_{A,C,L}(E_{M^j|A=0,C}(P(Y = 1|A, C, M^j, L)))$$

The first equality follows from the double expectation theorem. The second equality follows from the definition of $G^1_{0|C}$ (which is generated from the distribution of M^j_0 conditional on C, independently of A and L) and the 3rd equality since conditional on A, C and L, $G^j_{0|C}$ is independent of Y_{a,m^j}. The fourth equality follows as $M^j_0 \perp\!\!\!\perp A|C$. The fifth equality follows since $Y_{a,m^j} \perp\!\!\!\perp M^j|A = a, C, L$. The last equality follows by consistency.

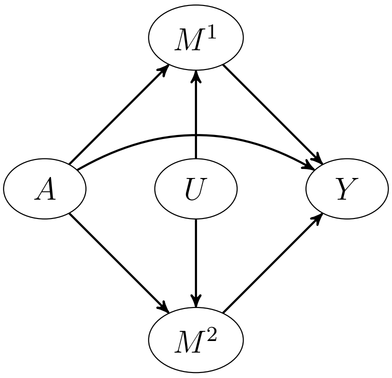

(a) M^1 and M^2 share an unmeasured common cause.

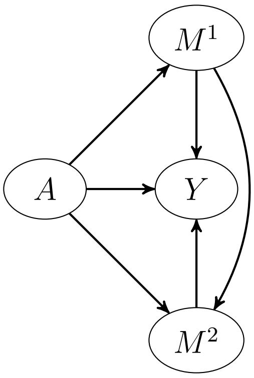

(b) M^1 affects M^2.

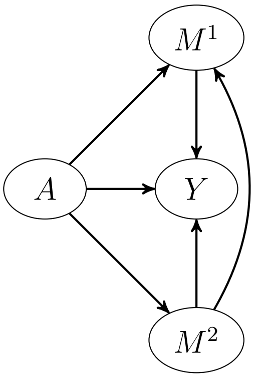

(c) M^2 affects M^1

Figure 2: Causal Diagrams. Confounders, C are omitted for display purposes.

Finally, in Figure 2(a), the unobserved variable U results in an open backdoor path biasing the $M^1 - Y$ and the $M^2 - Y$ relationships, even when conditioning on A. This again indicates for instance that condition 2. in the main manuscript does not hold. However, the condition, $(2^*) : Y_{a,m^j} \perp\!\!\!\perp M^j | A = a, L, C$ is satisfied in both cases by letting L represent the other mediator. That is even though U is unmeasured, $PAF^I_{A->M^1->Y}$ can still be identified via the formula above by letting $L = M^2$, and similarly $PAF^I_{A->M^2->Y}$ can be identified by letting $L = M^1$. This indicates we can identify interventional PS-PAFs under violations of condition (2) in the main manuscript due to either post treatment confounding induced by other mediators, or due to correlated error structure shared by differing mediators. Note that the mechanistic and separable PS-PAFs are non-identifiable in these instances.

Identification formula for separable pathway-specific population attributable fractions (PS-PAF)

Suppose that risk factor $A \in \{0, 1\}$ is divisible into separable components A_{M^j} and A_{O^j}, with causal DAG as represented by Figure 3 in the main manuscript, with $A = A_{M^j} = A_{O^j}$ with probability 1. We assume: $0 < P(A = 0|C = c)$, for all possible values of the covariate vector c. Then:

$$P(Y = 1|do(A_{M^j} = 0) = E_{A,C}(E_{M^j|A=0,C}(P(Y = 1|A, C, M^j)))$$

Proof

For any possible values of (c, a, m), where $\{C = c, A = a, M = m, y = 0\}$ has positive joint probability we have

$P(C = c, A = a, M^j = m, Y = 1|do(A_{M^j} = 0))$

$= P(C = c, A = a, A_{O^j} = a, M^j = m, Y = 1|do(A_{M^j} = 0))$

$= P(C = c)P(A = a|C = c)P(A_{O^j} = a|A = a)P(M^j = m|A_{M^j} = 0, C = c)$
$P(Y = 1|M^j = m, A_{O^j} = a, C = c)$

$= P(C = c)P(A = a|C = c)P(M^j = m|A_{M^j} = 0, A_{O^j} = 0, C = c)P(Y = 1|M^j = m, A = a, C = c)$

$= P(C = c)P(A = a|C = c)P(M^j = m|A = 0, C = c)P(Y = 1|M^j = m, A = a, C = c)$

where the first equality holds since $A_{O^j} = A$ with probability 1 under the intervention distribution, the second equality applys the g-computation formula from [2]

for the intervention distribution $do(A_{M^j} = 0)$. The condition $0 < P(A = 0|C = c)$ is necessary so that the conditional $P(M^j = m|A_{M^j} = 0, C = c)$ is well defined. The third equality follows by recognizing that $M^j \perp\!\!\!\perp A_{O^j}|A_{M^j}, C$ (an independence that can be read from the causal graph in the main manuscript) which implies that $P(M^j = m|A_{M^j} = 0, C = c) = P(M^j = m|A_{M^j} = 0, A_{O^j} = 0, C = c)$, and that $A_{O^j} = A$ with probability 1 which implies that $P(Y = 0|M^j = m, A_{O^j} = a) = P(Y = 0|M^j = m, A = a)$ and also allows us to drop the factor $P(A_{O^j} = a|A = a)$. The final equality follows since the event: $\{A_{M^j} = 0, A_{O^j} = 0\}$ and $A = 0$ are equal with probability 1.

It follows that:

$$P(Y = 1|do(A_{M^j} = 0)$$

$$= \sum_{a,m,c} P(C = c, A = a, M^j = m, Y = 1|do(A_{M^j} = 0)$$

$$= \sum_{a,m,c} P(C = c)P(A = a|C = c)P(M^j = m|A = 0, C = c)P(Y = 1|M^j = m, A = a, C = c)$$

$$= E_{A,C}(E_{M^j|A=0,C}(P(Y = 1|A, C, M^j)))$$

Equivalence of $PAF^S_{A->M^j->Y}$ and $PAF^M_{A->M^j->Y}$ when the causal directed acyclic graph (DAG) is separable

Note first that on an individual level the counterfactual: $Y_{a,M^j_0} = Y_{A=a,M^j=M^j_{A=0}}$ depends on A and M^j only through A_O and M^j (based on the separable graph), and as a result a potential outcome of Y associated with joint interventions on A and M^j can be rewritten as a potential outcome associated with the corresponding joint intervention on A_O and M^j. Noting that $A_O = a$, whenever $A = a$ with probability 1, it then follows that:

$$Y_{a,M^j_0} = Y_{A=a,M^j=M^j_{A=0}} = Y_{A_O=a,M^j=M^j_{A=0}} \tag{1}$$

with probability 1. Next note that Y depends on A_O and A_{M^j} only through their relationships with A_O and M^j. As a result potential outcomes of Y associated with joint interventions on A_O and A_{M^j} can be rewritten as potential outcomes associated with the corresponding joint intervention on A_O and M^j. Noting in addition that if $A_{M^j} = 0$, then $A = 0$ with probabilty 1, and so $M^j = M^j_{A=0}$ with probability 1, it follows that:

$$Y_{A_O=a,A_{M^j}=0} = Y_{A_O=a,M^j=M^j_{A=0}} \tag{2}$$

Combining (2) with (1), we see that on an individual level, $Y_{a,M^j_0} = Y_{A_O=a,A_{M^j}=0}$ with probability 1. Substituting the observed value, A for the risk factor, it follows that: $Y_{A,M^j_0} = Y_{A_O=A,A_{M^j}=0} = Y_{A_O=A_O,A_{M^j}=0} = Y_{A_{M^j}=0}$ with probability 1. Certainly then $P(Y_{A,M^j_0} = 1) = P(Y_{A_{M^j}=0} = 1)$. Now since, A_{M^j} is assumed to be a manipulable variable (that is a hypothetical real world intervention can be conceived of which sets A_{M^j} to 0 or 1 on a population level), the do-operator setting $A_{M^j} = 0$ is well defined and $P(Y_{A_{M^j}=0} = 1)$ is equal to $P(Y = 1|do(A_{M^j} = 0))$ indicating equivalence of the mechanistic and separable pathway-specific attributable fractions when causal separability conditions hold.

References

[1] Tchetgen EJT, VanderWeele TJ. On identification of natural direct effects when a confounder of the mediator is directly affected by exposure. Epidemiology. 2014;25:282.

[2] Robins JM, Richardson TS. Alternative graphical causal models and the identification of direct effects. Causality and psychopathology: Finding the determinants of disorders and their cures. 2010;84:103–158.
